# Comparison between Fluconazole with Oral Protexin Combination and Fluconazole in the Treatment of Vulvovaginal Candidiasis

**DOI:** 10.5402/2012/375806

**Published:** 2012-10-17

**Authors:** S. Nouraei, S. Amir Ali Akbari, M. Jorjani, H. Alavi Majd, M. Afrakhteh, A. Ghafoorian, H. Tafazzoli Harandi

**Affiliations:** ^1^Faculty of Nursing and Midwifery, Shahid Beheshti University of Medical Sciences, International Branch, Tehran, Iran; ^2^Department of Midwifery, Faculty of Nursing and Midwifery, Shahid Beheshti University of Medical Sciences, Tehran, Iran; ^3^Department of Pharmacology and Neuroscience, Research Center, Shahid Beheshti University of Medical Sciences, Tehran, Iran; ^4^Department of Biostatistics, Shahid Beheshti University of Medical Sciences, Tehran, Iran; ^5^Department of Obstetrics & Gynecology, Shahid Beheshti University of Medical Sciences, Tehran, Iran; ^6^Shahid Beheshti University of Medical Sciences, Tehran, Iran

## Abstract

*Background*. According to the limited studies reporting new treatments for vulvovaginal candidiasis, this study was designed to compare the combination of fluconazole and oral protexin with fluconazole in the treatment of vulvovaginal candidiasis. *Methods*. A double-blind clinical trial was conducted, involving 90 women who were referred to the gynecology clinic. Vulvovaginal candidiasis was diagnosed with itching, cheesy vaginal discharge, and any one of the following: dysuria, pH < 4.5, dyspareunia, vulvar erythema, or vulvar edema and if branched hyphae and Candida buds were visible after addition of KOH 10% in the culture and the result of cultivation in Sabouraud's dextrose agar medium was positive. Patients were randomly classified into two groups Absence of discharge, itching, and negative culture results 5–7 days after completion of treatment indicated treatment success. Data in this study were analyzed using the SPSS version 17.0 software. *Results*. The combinations, fluconazole-oral protexin and fluconazole-placebo, were equally effective in reduction of complaints and symptoms, but fluconazole-oral protexin combination elicited a better therapeutic response (*χ* ^2^ = 0.01, *P* = 6.7). In addition, fluconazole-oral protexin combination treatment demonstrated better recovery time (*t* = −2.04, *P* = 0.04). *Conclusion*. This study demonstrated that complementary treatment with probiotic Lactobacillus increased the efficacy of fluconazole in treatment of vulvovaginal candidiasis. Further research is recommended.

## 1. Introduction

Vaginitis is the most common gynecological problem, for which women seek treatment [1, 2]. Four types of infectious vaginitis are commonly found in women—candidiasis, trichomoniasis, bacterial vaginosis, and gonococcal infections [3]. Among these, candidiasis is the second most common vaginal infection. Seventy-five percent of women suffer from vulvovaginal candidiasis at least once during their lives, almost 45% of women experience the disease twice or more annually and approximately 5% of women are diagnosed with chronic and recurrent infections [4–6].

The disease is most prevalent among women aged 25–35 years. A variation in the incidence of vulvovaginal candidiasis in various communities has been reported in Iran. The prevalence of vulvovaginal candidiasis in Mashhad was reported to be 43% in 2005 [7]; in 1997, it was reported to be 19.8% in Kerman, 22.3% in Kashan, and 26.7% in Sari [8].

The etiology of vulvovaginal candidiasis also varies. *Candida albicans* is the most commonly found genera in the genitalia in 80–90% of cases of vulvovaginal candidiasis. *C. glabrata* is the second most common cause of the disease and is found in 5%–15% of cases [6, 7]. Use of antibiotics, oral contraceptives, corticosteroids, and immunosuppressive drugs increases the risk of contracting this disease; pregnancy and diabetes in combination with normal changes in the vaginal flora are also risk factors for vulvovaginal candidiasis [2, 7, 9]. Although this disease is not life-threatening, symptoms such as itching, irritation, pain during intercourse, and secretion cause physical discomfort to occur, and the treatment is expensive. In addition, mental and psychological damage can occur, especially in chronic, untreated, and recurrent cases, because of the burden of living with these symptoms. Vulvovaginal candidiasis may also influence sexual functions and disrupt an individual's sex life [4, 6, 10].

 Several types of antifungal agents exist for vulvovaginal candidiasis, including nystatin and any pharmaceutical agent containing an azole product, such as miconazole, clotrimazole, or fluconazole, some of which come in the form of suppositories, creams, and vaginal pills [11]. Use of vaginal medication can be difficult because it may cause local irritation or stimulation. Once the symptoms reduce, treatment is often left incomplete before the disease is eradicated [10, 12]. 

Clotrimazole is the first-line treatment for vulvovaginal candidiasis. Fluconazole is used in cases with no response to clotrimazole treatment [12]. Fluconazole is an antifungal agent that includes azole and has the same effect as vaginally administered products in the treatment of vulvovaginal candidiasis [1]. Fluconazole is administered orally as a unit dosage [2]. This administration method avoids the discomfort during vaginal treatment. It has the additional advantage of treating mycosis in the gastrointestinal tract [11]. Nevertheless, recurrence is possible in patients treated with fluconazole. 

Some researchers have suggested that vulvovaginal candidiasis leads to destruction of the vaginal flora; therefore, the efficacy of *Lactobacilli* (probiotics) in avoiding vaginal infections has been investigated [4, 13]. Probiotics are live microorganisms that have beneficial effects on the health of the host [5, 14]. *Lactobacilli* are natural inhabitants of the vulva and vaginal flora. They play a fundamental role in suppression of potential pathogens. *Lactobacilli* administered within the genital tract act as prophylaxis, improving and strengthening the genital microflora and defending against bacterial infections [15]. Two major groups of these microorganisms include probiotic *lactobacillus* and *Bifidobacterium* [16, 17].

Some studies have suggested that the effects of probiotic *Lactobacilli* in the treatment of candidiasis are vulvovaginal [4, 15]. However, the effects are as yet unproven and remain controversial [9, 18, 19]. Suggested beneficial effects of probiotics include anticholesterol and antioxidative activity, reduced risk of colon cancer and diarrhea caused by rotavirus and use of antibiotics, reduced incidence of infection due to *Helicobacter pylori*, reduced constipation, alleviation of symptoms of inflammatory bowel disease, fewer urinary tract, vaginal, and respiratory infections; and treatment and prevention of allergy symptoms [9, 14, 17, 20]. The high prevalence of vulvovaginal candidiasis, the multiple complications associated with use of chemical agents, the increase in microorganism resistance to antibacterial drugs, and the need for a diet to improve the efficacy of existing therapies all contributed to the decision to conduct this research. This study was designed to compare the effects of combination treatment with fluconazole-oral protexin and fluconazole-placebo in the treatment of vulvovaginal candidiasis.

## 2. Materials and Methods

### 2.1. Subjects

A double-blind clinical trial was conducted, involving 90 women who were referred to the obstetrics unit of the clinic affiliated with Shahid Beheshti University of Medical Sciences in Iran. Initially, 102 women were enrolled in the study according to the following specifications: 18–40 years of age; married; in a monogamous relationship; not pregnant or lactating; not menstruating at the time of referral; receiving clotrimazole; absence of condition improvement; not using any vaginal medication, antibiotics, immunosuppressive drugs, or exogenous hormones, including oral contraceptives, during the 2 weeks before study initiation; abstaining from intercourse or vaginal douche in the previous 24 h; absence of other trichomonal vaginal infections or bacterial vaginosis; absence of known systemic disease such as diabetes or other autoimmune disease; showing positive potassium hydroxide (KOH) samples in culture. Twelve subjects were excluded because they demonstrated allergic reactions to fluconazole or protexin, conceived during treatment, commenced treatment with antibiotics or any other antifungal drug, began menstruation, used a vaginal douche during the treatment period, or had intercourse without a condom during the treatment period. 

The research tools included

1. *An inclusion criteria questionnaire.* This questionnaire included information regarding age, marital status, pregnancy and lactation status, history of previous disease, history of previous drug use, and contraception methods.
2. *Demographics and obstetric history questionnaire*. A questionnaire gathered information about obstetric history and patient demographics. Questions regarding the education level, occupation, age, duration of marriage, number of pregnancies, number and type of deliveries, number of abortions and curettages, menstrual status, type of menstrual products used during menstruation, and sex and bathing habits were included.
3. *Observation checklist for the first visit (before treatment).* The first part of this questionnaire was related to complaints at the first visit, including vaginal discharge, itching, dysuria, and pain during intercourse and urination. In the second part, pH score and symptoms during the first examination, including vulvitis and vulvar redness, vaginal discharge, and vulvar edema, were reviewed. In addition, information regarding the existence of branched hyphae and *Candida* buds in the wet slide, culture result, and the type of *Candida* infection is included.
4. *Observation checklist for the second visit (after treatment).* This checklist was similar to the first, with the addition of questions about treatment-related complications.
5. *Daily checklist for patients*. A daily checklist for completion by participants was also used in this study. Throughout the treatment period, patients took daily notes of their physical symptoms and complaints based on the observation checklist. The date on which symptoms reduced, completely disappeared, or relapsed was also recorded.
6. A microscope.
7. A pH indicator paper.
8. Agar medium.

To determine the validity of the questionnaire and observation checklist, content validity testing was performed. To determine the reliability of the observation checklist, the kappa coefficient was used to ensure agreement among raters. Thus, 10 patients referred to the clinic for vulvovaginal candidiasis were examined and questioned about the checklist simultaneously by the primary researcher and a clinic professional of the same rank. The kappa coefficient was then calculated to determine the reliability of the checklist. The minimum acceptable coefficient was 0.80. 

To ensure the reliability of the pH paper (Merck, Darmstadt, Germany), 5 samples were taken from the same person and their pH values were assessed. Uniformity of the obtained results confirmed the reliability of this tool.

To ensure the reliability of the microscope (Nikon, Tokyo, Japan), accurate calibration of the device was performed. Then, 5 slides were prepared from a single sample and investigated using the microscope. Uniformity of results was compared with those from another standard microscope; the results were the same. Thus, the reliability of the microscope used in this study was confirmed.

Prepared slides were codified in the *Laboratory* by an experienced microbiologist. Codes of some slides were then changed, and the slides were reviewed again by the same person. Uniformity of the obtained responses confirmed reliability of the *Laboratory* results. All slides were reviewed using the same microscope by the same *Laboratory* science specialist.

To assess the reliability of the agar medium (Darvash Ltd., Tehran, Iran), several cultures were prepared from the same patient. Cultures were then reviewed by a *Laboratory* science expert. Reliability of the agar medium was determined through the uniformity of responses.

Participants who met the inclusion criteria and conformed to the specifications of the research unit were instructed about this paper and its goals. Verbal consent was obtained from all participants prior to the examination and sampling. 

Participants were first placed in the lithotomy position. A sterile speculum without lubricant was placed, and the vagina and crevices were evaluated for abnormal inflammation and secretions in terms of color, consistency, and odor. Samples were taken from the upper wall of the vagina. 

Samples were placed on two slides and a plate containing agar medium and checked for *Trichomonas vaginalis*, bacterial vaginosis, and *C. albicans*. For microscopic investigation, 1 or 2 drops of normal saline were added to the first slide sample, which was investigated for key cells and *T. vaginalis*. In cases where flagellant *T. fungus* was identified, samples were excluded. One drop of solution (KOH 10%) was added to the second slide for investigation in terms of *Candida* hyphal category and amine odor. Samples in which bacterial vaginosis infection was identified were excluded. 

Culture samples were also transferred to Taleghani Hospital *Laboratory* daily, and the culture results were analyzed within 24–48 h of sampling. *C*. *albicans* was distinguished from other types of *Candida* using a germ tube test. In addition, vaginal pH was determined using the pH indicator paper. Finally, if symptoms of itching, cheesy vaginal discharge, and any one of dysuria, pH < 4.5, dyspareunia, vulvar erythema, or vulvar edema were present, or if branched hyphae *Candida* buds were visible after KOH 10% was added in culture, or if a positive result was observed in culture using Sabouraud's dextrose agar medium, vulvovaginal candidiasis was diagnosed. 

After written consent had been obtained, 90 of the original 102 participants in this study were randomly classified into two treatment groups—a fluconazole-placebo group and a fluconazole-protexin combination group. The two groups were similar in terms of age, educational level, age at marriage, marriage duration, age at first pregnancy, obstetric status, menstrual status, contraception methods, and health status. Subjects in these groups were codified and the researcher was blinded to the groupings until the end of the study period.

All participants were provided with the relevant instructions and recommendations for drug usage. In addition, an educational pamphlet regarding their condition was prepared and given to all patients. In the fluconazole-placebo group, fluconazole capsules (2 × 150 mg) and 20 placebo capsules were distributed within an interval of 72 h (3 days). Two placebo capsules were administered per day after meals in the morning and evening. The same protocol was used in the fluconazole-oral protexin group, except that instead of placebo 20 protexin capsules were distributed. Patients were referred to the medical center 5–7 days after the start of treatment for reevaluation of clinical and *Laboratory* symptoms. At this point, the absence of discharge and itching as well as negative culture results indicated treatment success.

All data were analyzed using descriptive statistics (means and standard deviations) and inferential statistics (*t*-test, chi-square test, Mann-Whitney *U* test, Fisher's exact test, and McNemar's test) using the SPSS software, version 17.0 (SPSS, Inc., Chicago, IL, USA).

Written permission to conduct this study was obtained from the International Branch of the School of Nursing and Midwifery of Shahid Beheshti University of Medical Sciences. This study was registered in the Trial Center of Iran (number IRCT201106206807N3). Permission was obtained from the president of the participating clinic.

## 3. Findings

No significant differences were found between the subjects in terms of average age, age at marriage, marriage duration, age at first pregnancy, number of pregnancies, cesarean delivery, natural labor, abortion, or curettage in the two treatment groups according to the results of the *t*-test and Mann-Whitney *U* test (Table 1). Majority of the subjects in both treatment groups did not use swimming pools, public baths, tubs, or genitalia washing gels. Fisher's exact test showed no significant difference between the two treatment groups for these factors (*P* = 0.73).

Of those participants with 100% positive culture for *Candida*, *C. albicans* infection was confirmed in 91.2% of subjects in the fluconazole-placebo group and 93.4% of subjects in the fluconazole-oral protexin group before the start of treatment. The two groups were similar in this regard (*P* = 0.69).

In the present study, the most common complaints in both treatment groups were itching and vaginal discharge. Pain during intercourse and dysuria were the next most common problems. Pain during urination was the symptom with the lowest incidence in both treatment groups.

Fisher's test and the Chi-square test revealed no significant differences between the two treatment groups regarding posttreatment complications (*P* = 0.22). Therefore, both treatment methods were deemed to have been effective. However, a significant difference in the effect of treatment on dysuria (*P* = 0.02,   *χ* ^2^ = 4.86)  was found. The fluconazole-oral protexin combination was more effective in treatment of dysuria than fluconazole-placebo (Table 2).

Vulvar edema was the most common symptom followed by vulva inflammation and redness. No significant difference was observed between the two treatment groups in terms of vulvar inflammation and redness (*P* = 0.18) or vulvar edema after the treatment (*P* = 0.53), according to Fisher's test and chi-square test (Table 3).

*C. albicans* was detected in most participants in both treatment groups before the treatment. After the treatment, *C. albicans *was detected in only 8.8% of participants in the fluconazole-placebo treatment group and 4.4% in the fluconazole-oral protexin treatment group. McNemar's statistical test showed a significant difference for this parameter between the two treatment groups before treatment; however, after treatment, results for the two groups were identical. Therefore, both the treatment methods were deemed to have been effective in eradicating *C. albicans *(Table 4).

A significant difference was found between the ratio of participants in the two treatment groups in terms of treatment success. The results demonstrated that the combination of fluconazole and oral protexin was a more efficient treatment (*P* = 0.01,  *χ* ^2^ = 6.7) (Table 5).

The average recovery time in the fluconazole-oral protexin group was shorter than in the fluconazole-placebo group (5.36 ± 1.85 versus 6.22 ± 2.0). A significant difference was observed between the two treatment groups in terms of recovery time; patients treated with fluconazole-oral protexin recovered faster (*t* = − 2.04, *P* = 0.04).

Most subjects in both groups experienced no side effects. Nausea was the most frequently reported symptom in the fluconazole-placebo treatment group (6.7%).

## 4. Discussion

The results of this study showed that complementary treatment with probiotic *lactobacillus* increased the efficacy of treatment of vulvovaginal candidiasis with fluconazole.

 In the present study, the most common complaints in both groups were itching and vaginal discharge. Vulvar edema, inflammation, and redness were the most common symptoms. Oriel et al. reported that vaginal itching with or without discharge is seen in 50% of patients, whereas only 30% of patients with positive culture experienced vaginal discharge alone [21]. Ventolini et al. stated that itching without discharge is only predictive of vulvovaginal candidiasis in 38% of patients [22]. Tehrani et al. reported a prevalence of abnormal secretions in 55.9% of patients, itchy vagina in 32.8%, unpleasant odor of discharge in 36.5%, changes in discharge in 50.8%, pain or irritation when urinating in 12.2%, and vaginal irritation in 20.1% [23].

In this study, pH values < 4.5 were observed in 91.2% of subjects in the fluconazole-placebo treatment group and 93.4% of subjects in the fluconazole-oral protexin treatment group before the treatment. Speroff and Fritz demonstrated that *Candida* infection typically occurs in the postmenstrual phase, because during menstruation vaginal pH is high enough to allow the *Candida* organism to colonize [24]. In a study by Kamali et al., pH levels > 3.9 were observed in the vaginal discharge of all subjects before treatment [25]. Runeman et al. claimed that high vaginal pH in healthy women and women with vulvovaginal candidiasis is natural at approximately 4.5 [26]. 

Rönnqvist et al. found that *Lactobacilli *administered in the genital pathway as a prophylaxis improved and strengthened the defenses of the genital microflora against bacterial infection. *Candida* alters the normal vaginal flora. During probiotic treatment, probiotic metabolites reach the entire body, including the vagina, through the bloodstream and cause changes in the normal vaginal flora [15].

Ehrström et al. asserted the value of probiotic treatment of vulvovaginal symptoms for 1–3 days after the second menstrual cycle [5]. In that study, women in the intervention group (taking probiotic supplement) complained significantly less discharge than women in the placebo group (*P* = 0.03 and *P* = 0.04, resp.). Irritation and itching after treatment with probiotic vaginal capsules in the intervention group were slightly higher than in the placebo group (NS). This study found no difference between participants in whom *Lactobacilli* had colonized and those in whom *Lactobacilli* had not colonized in terms of vaginal pH, reported symptoms, or clinical treatment [5].

In a study by Martinez et al., all participants in both treatment groups (fluconazole-protexin and fluconazole placebo) complained of vaginal discharge along with at least one symptom among the following: itching, vaginal irritation, dyspareunia, and dysuria before treatment. After treatment, only 10.3% of subjects in the fluconazole-protexin treatment group and 34.6% of subjects in the fluconazole-placebo treatment group complained of vaginal discharge and at least one of these symptoms (*P* = 0.03) [4].

In the study of Tafazzoli Harandi, comparing the effects of metronidazole and a metronidazole-probiotics combination in the treatment of bacterial vaginosis, no significant difference was found between the two treatment groups in terms of amount of vaginal discharge, odorous discharge, dysuria, and itching after treatment. However, results for dysuria were at the threshold of significance, which indicates that the effect of metronidazole and probiotics on dysuria was greater than the effect of metronidazole alone (*P* = 0.06) [27]. However, both treatment methods were effective in alleviating the symptoms of patients.

Reid et al. found a 12% improvement in vaginal symptoms in patients treated with orally administered *L. rhamnosus* and *L. fermentum* (30%) compared with placebo [28]. Falagas et al. suggested that *Lactobacilli* secrete lactic acid and other substances that maintain low vaginal pH, thereby preventing excessive growth of pathogens in the vagina [9]. The study of Hilton et al. noted a significant improvement of symptoms in all women with *Candida* who were treated with vaginal suppositories of *L. acidophilus*. In addition, erythema and vaginal discharge decreased during the study period in treated participants [29].

Boris et al. identified a connection between *L. acidophilus*, *L. casei*, and genital *lactobacillus in vitro* with *C. albicans*. This connection reduces the adhesion of *C. albicans* to the vaginal epithelial cells, thereby reducing or preventing development of vulvovaginal candidiasis. The accumulation of *Lactobacilli* in patients with *Candida* can prevent vaginal infections, especially vulvovaginal candidiasis, by preventing binding of the bacteria to the receptors of the vaginal epithelium [30]. Reid et al. confirmed that the biological surfactants produced by *L. fermentum* RC-14 inhibit adhesion of *C. albicans* to the vaginal walls [28]. The study of Hilton et al. found negative cultures in 4 out of 5 women with *C. albicans-*positive culture in the vagina prior to treatment after administration of *Lactobacilli* [29].

Shalev et al. identified a positive culture for *L. acidophilus* before treatment in 20% of participants receiving 150 mL of yogurt containing living bacteria (group I) on a daily basis versus 31% in the group receiving 150 mL daily of pasteurized yogurt (group II). After 1 month of treatment, positive cultures for *L. acidophilus* were found in 71% of participants in the first group and 27% in the second group. Thus, the number of women with positive cultures in the first group increased after the first and second months of the study. These numbers were significantly higher than those in the second group. Although a progressive decrease was observed in the Candida-positive cultures in both groups, no significant difference in the percentage of women with positive cultures was found between the two groups 1 and 2 months after the start of the study [31].

Martinez et al. found positive cultures for *C. albicans* before treatment in both treatment groups in their study. After the treatment, only 10.3% of subjects in the group treated with fluconazole-protexin and 38.5% of subjects in the group treated with fluconazole-placebo had Candida-positive cultures (*P* = 0.01) [4]. Strus et al. suggested that *Lactobacilli* that secrete high levels of H_2_O_2_ enhance the growth of *C. albicans* and inhibit it faster than other species [32].

According to the results of the current study, vulvovaginal candidiasis was successfully treated in 60% of subjects in the fluconazole-placebo treatment group and 84.4% of subjects in the fluconazole-oral protexin group. Thus, a significant difference between these treatments in terms of treatment success was observed. The combination of fluconazole and oral protexin performed better in terms of efficiency.

Reid conducted three hypotheses explaining the activity by which probiotics can strengthen the performance of antibiotics. Firstly, probiotics reduce the risk of antibiotic-driven infections in the intestine and vagina. Secondly, probiotics secrete antibacterial substances that locally reduce pathogen populations in the mucus, enabling antibiotics to work better. Finally, probiotics generally improve the integrity of the mucosal barrier, which helps to eradicate pathogens in the mucus [14].

In their study, Murina et al. claimed that the use of fluconazole plus probiotic in the treatment of *Candida* infections could have a synergistic effect, maintaining homeostasis and balance in the vaginal flora [33].

In their study, Ehrström et al. noted a success rate of treatment after the first menstrual period of 78% in the intervention group (probiotic supplement) and 71% in the placebo group. After the second menstrual period and 6 months after the end of treatment no difference in the value of clinical treatment was observed between the intervention and placebo groups [5].

Martinez et al. found a positive response rate of 89.7% in the group treated with fluconazole-protexin. The success rate was 61.5% in the group treated with fluconazole-placebo. These results indicate that probiotics can be successfully used in treatment of *Candida* infections. Treatment with probiotics was successful because of inhibition of the growth of *C. albicans* in the vagina and strengthening of the immune system in the vagina, small bowel, and colon and reduction of fungi in the rectum and vagina [4].

In the current study, no complications occurred in most subjects in both treatment groups. Falagas et al. claimed that in general probiotics can be considered safe. Probiotics can be especially useful when the use of antifungal drugs is contraindicated or may cause side effects [9].

Ehrström et al. reported itching in one subject (1.6%) in the group receiving probiotic supplementation (intervention group) and 5 women (13.2%) in the placebo group. One woman (1.6%) in the intervention group experienced slight vaginal bleeding, while vulvar swelling and redness developed in one subject (1.6%) after treatment with placebo. One subject in the placebo group complained of headache [5]. In the study of Martinez et al., no specific complications were expressed in groups treated with fluconazole-protexin and fluconazole-placebo [4].

Thus, the results of this study are consistent with those of many studies. Differences between the results reported here and those of other studies may be attributed to use of *Lactobacilli* alone in some studies, differences in Kant *lactobacillus* colonization, use of vaginal *Lactobacilli*, and administration of drugs other than fluconazole. Further studies are essential to confirm the effect of probiotics in vaginitis, especially vulvovaginal candidiasis.

This study demonstrated that complementary treatment with probiotic *lactobacillus* increased the efficacy of fluconazole in treatment of vulvovaginal candidiasis. Further research is recommended.

## Figures and Tables

**Table 1:** Comparison of means and standard deviations of variables in subjects in the two treatment groups (fluconazole-placebo and fluconazole-oral protexin).

Groups	Fluconazole-placebo	Fluconazole-oral protexin	Test results	
Variables	Index			
	Meanand standard deviation	Meanand standard deviation	Statistic	*P* value
Marriage age (years)	20.82±4.19	20.09 ± 2.94	*t* =−0.96	0.33
Marriage duration (years)	9.44 ± 6.11	10.40 ± 5.83	*t* =0.75	0.45
Age at first pregnancy (years)	21.78 ± 4.00	21.65 ± 3.00	*t* =−0.16	0.86
Number of pregnancy	1.71 ±1.34	1.82 ± 1.40	Mann Whitney 961	0.67
Number of delivery	1.60 ± 1.26	1.64 ± 1.31	Mann Whitney 990	0.85
Cesarean delivery	1.83 ± 84%	1.50 ± 71%	Mann Whitney 992	0.56
Natural delivery	45% ±71%	58% ±74%	Mann Whitney 991	0.48
Number of abortions	0.11 ± 0.38	0.18 ± 0.38	Mann Whitney 902	0.22
Number of curettage	0.07 ± 0.33	0.11 ± 0.22	Mann Whitney 900	0.23

**Table 2:** Absolute and relative frequency distribution of subjects with vulvovaginal candidiasis according to complaints before and after treatment in the two treatment groups (fluconazole-placebo and fluconazole-protexin).

Groups	Fluconazole + placebo					Fluconazole + protexin					
Complaints	Index										Intergroup comparison
	Before treatment		After treatment		Intergroup	Before treatment		After treatment		Intragroup
	Total	Percent	Total	Percent	comparison	Total	Percent	Total	Percent	comparison
Vaginal secretion	45	100	5	11.2	—	45	100	2	4.4	—	NS Fisher
Itching	45	100	7	15.6	—	45	100	7	15.6	—	NS Chi-square
Dysuria	23	51.1	12	26.7	0.001	21	46.7	4	8.9	0.001	*P* = 0.02
Pain during intercourse	28	62.2	6	13.4	0.001	22	48.9	8	17.8	0.001	NS Chi-square
Pain when urinating	9	20	3	6.7	0.031	11	24.4	7	15.6	0.125	NS Fisher

**Table 3:** Absolute and relative frequency distribution of subjects with vulvovaginal candidiasis according to symptoms before and after treatment in the two treatment groups (fluconazole-placebo and fluconazole-protexin).

Groups	Fluconazole + placebo					Fluconazole + protexin					
Symptoms	Index										Intergroup comparison
	Before treatment		After treatment		Intergroup	Before treatment		After treatment		Intragroup
	Total	Percent	Total	Percent	comparison McNemar	Total	Percent	Total	Percent	comparison McNemar
Vulva inflammation and redness	10	22.2	3	6.7	0.016	17	37.8	7	15.6	0.002	NS Fisher
Vulva edema	18	40	7	15.6	0.001	15	33.4	5	11.1	0.002	NS Chi-square
pH < 4.5	41	91.2	42	93.4	0.50	42	93.4	44	97.8	0.50	NS Chi-square

**Table 4:** Absolute and relative frequency distribution of subjects with vulvovaginal candidiasis according to the wet slide test, culture results, and type of Candida before and after treatment in the two treatment groups (fluconazole-placebo and fluconazole-protexin).

Groups	Fluconazole + placebo					Fluconazole + prebiotics					
	Indicators										Intergroup comparison
	Before treatment		After treatment		Intragroup	Before treatment		After treatment		Intra-group
	Total	Percent	Total	Percent	comparison	Total	Percent	Total	Percent	comparison
Existence of *hypha* in wet slide	45	100	12	26.7	—	45	100	8	17.8	—	NS Chi-squared
Culture results	45	100	8	17.8	—	45	100	3	6.7	—	NS Fisher
*Albicans *	41	91.2	4	8.8	0.001 McNemar	42	93.4	2	4.4	0.001 McNemar	—

**Table 5:** Absolute and relative frequency distribution of subjects with vulvovaginal candidiasis according to treatment response in the two treatment groups (fluconazole-placebo and fluconazole-protexin).

Groups	Fluconazole + placebo		Fluconazole + protexin		Sum	
Therapeutic response	Total	Percentage	Total	Percentage	Total	Percentage
Treatment success	27	60	38	84.4	65	72.2
Treatment failure	18	40	7	15.6	25	27.8
Sum	45	100	45	100	90	100
