# Corrigendum to “Comparison between Fluconazole with Oral Protexin Combination and Fluconazole in the Treatment of Vulvovaginal Candidiasis”

**DOI:** 10.1155/2017/9375678

**Published:** 2017-11-27

**Authors:** S. Nouraei, S. Amir Ali Akbari, M. Jorjani, H. Alavi Majd, M. Afrakhteh, A. Ghafoorian, H. Tafazzoli Harandi

**Affiliations:** ^1^Faculty of Nursing and Midwifery, Shahid Beheshti University of Medical Sciences, International Branch, Tehran, Iran; ^2^Department of Midwifery, Faculty of Nursing and Midwifery, Shahid Beheshti University of Medical Sciences, Tehran, Iran; ^3^Department of Pharmacology and Neuroscience, Research Center, Shahid Beheshti University of Medical Sciences, Tehran, Iran; ^4^Department of Biostatistics, Shahid Beheshti University of Medical Sciences, Tehran, Iran; ^5^Department of Obstetrics & Gynecology, Shahid Beheshti University of Medical Sciences, Tehran, Iran; ^6^Shahid Beheshti University of Medical Sciences, Tehran, Iran

In the article titled “Comparison between Fluconazole with Oral Protexin Combination and Fluconazole in the Treatment of Vulvovaginal Candidiasis,” [1] there was an error in the Abstract section, where “(*χ*^2^ = 0.01, *P* = 6.7)” should be corrected to “(*χ*^2^ = 6.7, *P* = 0.01)”.
